# Quantifying the Foregone Benefits of Intelligent Speed Assist Due to the Limited Availability of Speed Signs across Three Australian States

**DOI:** 10.3390/s22207765

**Published:** 2022-10-13

**Authors:** Sujanie Peiris, Stuart Newstead, Janneke Berecki-Gisolf, Brian Fildes

**Affiliations:** Accident Research Centre, Monash University, 21 Alliance Ln, Clayton, VIC 3800, Australia

**Keywords:** intelligent speed assist, advisory, roads, infrastructure, speed signs, speed sign sensing

## Abstract

By being able to communicate the speed limit to drivers using speed sign recognition cameras, Intelligent Speed Assist (ISA) is expected to bring significant road safety gains through increased speed compliance. In the absence of complete digital speed maps and due to limited cellular connectivity throughout Australia, this study estimated the forgone savings of ISA in the event that speed signs are solely relied upon for optimal advisory ISA function. First, speed-related fatalities and serious injuries (FSI) in the Australian states of Victoria, South Australia, and Queensland (2013–2018) were identified, and published effectiveness estimates of ISA were applied to determine the potential benefits of ISA. Subsequently, taking into account speed sign presence across the three states, the forgone savings of ISA were estimated as FSI that would not be prevented due to absent speed signage. Annually, 27–35% of speed-related FSI in each state are unlikely to be prevented by ISA because speed sign infrastructure is absent, equating to economic losses of between AUD 62 and 153 million. Despite a number of assumptions being made regarding ISA fitment and driver acceptance of the technology, conservative estimates suggest that the benefits of speed signs placed consistently across road classes and remoteness levels would far outweigh the costs expected from the absence of speed signs. The development and utilisation of a methodology for estimating the foregone benefits of ISA due to suboptimal road infrastructure constitutes a novel contribution to research. This work provides a means of identifying where infrastructure investments should be targeted to capitalise on benefits offered by advanced driver assist technologies.

## 1. Introduction

Speeding is the largest behavioural contributor to road traffic deaths and injuries in Australia and other developed countries [1], with speeding (i.e., travelling above the sign-posted speed limit) being cited as the most common road safety infringement [2]. Speed regulation remains a highly controversial area of road safety, with significant media resistance to positive speed management strategies, policies, and practice [3,4,5], even though poor speed compliance contributes to both crash risk and severity [6]. Due to the limited success of many speed reduction strategies and targeted interventions, advanced driver assist technologies, such as Intelligent Speed Assist (ISA), have the potential to influence driver speeding and are being considered as viable speed management interventions.

Given the prevalence of speeding in Australia and overseas, the ability to mitigate speeding is likely to have significant road safety benefits. In certain jurisdictions, such as Queensland, up to 94% of drivers are estimated to engage in low-level speeding (i.e., travelling up to 10 km/h above the limit) [7], while in other states this estimate is 78% or 88% [8,9]. It is therefore not surprising that a large proportion of fatal and serious injury crashes involve individuals who are exceeding the speed limit. Estimates suggest that, in NSW, up to 38% of all casualty crashes and 76% of fatal crashes could be attributed to low-level speeding [9]. These figures are reinforced by those in other areas of the world, with low- and medium-level speeding found to correspond to a major fraction of fatal crashes in Europe [10].

The exact contribution of speeding to crashes, however, is a contentious topic as it is difficult to quantify. Crash databases in most jurisdictions, both in Australia and internationally, do not record detailed information regarding speed involvement as a contributing or causative factor to the crash. Of those that do, reference to speed involvement is only general, and caution should be exercised in using these data to ascertain the proportion of crashes that involve speeding as a causal factor [11]. This is because crash data are typically compiled by attending police officers who do not reconstruct crashes to determine pre-crash speed estimates. Such estimates should be based on Newtonian mechanics, considering vehicle damage profiles and resting positions of the vehicles, and are thus resource intensive.

Estimates of speed involvement using the GIDAS study [12], which examined 10,742 fully documented and reconstructed crashes (Germany, 2011–2016), identified that 24.2% of fatal crashes, 18.5% of fatal and serious injury crashes, and 15.2% of all injury crashes involved speed as a contributing factor. Overall estimates suggest that 10 to 15% of all crashes and 30% of all fatal crashes in Europe are a direct result of speeding or inappropriate speed [13]. In the USA during 2017, speeding was implicated in 26% of all road trauma fatalities [14], but this figure was as high as 35% in some states [15]. In New Zealand, up to 30% of fatal crashes were attributed to speeding, although this figure was estimated to be as high as 60% when corrective factors from another study were applied [11]. Estimates from event data recorders analysed from South Australian crashes suggest that speeding contributes to 18% of all serious and fatal road trauma [16], but the contribution of speed to fatalities and serious injuries from crashes in Victoria is thought to be as high as 30% and 25%, respectively [17]. While scientific evidence that links fatal and serious injury crashes with inadvertent or unintentional speeding is sparse, it is highly likely that at least a proportion of those crashes attributed to speeding involve those inadvertently speeding [1,18,19]. Given the relationship between travel speed and crash risk, and travel speed and crash severity, the ability to mitigate speed-related crashes, whether it be low- or high-level speeding, inadvertent, or intentional, is likely to have significant road safety gains [20,21].

Given the limited success of physical speed countermeasures such as chicanes and speed bumps [22,23,24,25,26,27,28,29], advanced in-vehicle driver assist technologies that can influence travel speed, such as ISA, are expected to bring significant reductions in road trauma [30,31,32,33] by informing drivers of the posted speed limit for the road they are travelling on. ISA technology commonly uses vehicle-mounted sign recognition cameras and a GPS-linked speed limit database to display the legal speed limit on the dashboard. There are, at present, three forms of ISA that are designed to aid drivers in observing the speed limit: (1) advisory ISA, which notifies the driver when the travel speed of the vehicle exceeds the legal speed limit through an audio or visual warning; (2) voluntary or supportive ISA, which enables the driver to choose if the vehicle should adjust to the speed limit if/when the speed limit is exceeded; and (3) mandatory or limiting ISA, which physically regulates the vehicle’s travel speed based on the speed limit detected.

It is expected that if drivers are constantly aware of the speed limit, and notified if and when it is being exceeded, that they are more likely to adhere to the speed limit. Based on this premise, it is estimated that the most intervening variant of ISA (limiting ISA, if fitted to every vehicle) could reduce crash likelihood by up to 30% and road deaths by 20% in Europe [34], while these figures could be as high as 30% and 28%, respectively, in Australia [35]. Despite the large safety gains expected from supportive or limiting ISA, advisory ISA, because of its limited intrusiveness and greater potential for public acceptance, is the most widely promoted form of ISA at present, with all newly manufactured vehicles (from May 2022) in Europe required to be fitted with the technology [36]. The mandate is unlikely to be unique to Europe, with ANCAP, Australia’s independent vehicle safety testing authority, promoting ISA fitment and assessing vehicles based on the presence and performance of the technology [37].

The ability of ISA to function, however, is dependent on the technology being able to identify local speed limits using cellular connectivity (which allows the vehicle to access digital speed maps) or, in its absence, being able to access physical speed signs which vehicle-mounted cameras can detect using embedded sensors [38]. The European Commission has specified that ISA systems that employ a combination of camera systems, possess a Global Navigation Satellite System (GNSS), and use up-to-date digital maps are *state of the art*, having *the greatest real-world performance and reliability* [39]. In recognition of the limitations associated with accessing digital speed maps, and in support of the recent technology mandate in the EU, all member states of the EU were encouraged to facilitate the correct placement of numerical speed limit signs on streets and roads with clear indications of when a speed zone starts and ends [39]. Cellular networks enable continuous data connectivity, supporting a number of autonomous driver functions by delivering information about traffic and road conditions over the wireless network. In the case of ISA systems, access to mobile data would enable vehicles to acquire base map information and receive live updates regarding posted speed limits. Such connectively, however, is absent or limited in many regions of Australia, and, as an alternative, speed sign infrastructure is required for ISA to function [40]. In particular locations, such as in remote and regional Australia, cellular connectively is absent [41] and vehicles fitted with ISA are solely reliant on physical speed signs. Reflecting this, existing ANCAP test protocols for ISA do not specify the need for vehicles to be able to access digital speed maps, provided that the speed recognition cameras can detect speed signs and relay the appropriate information to the driver [42].

### 1.1. Speed Signage and ISA

The lack of regulation regarding speed sign availability (frequency and/or placement) and consistency (position, size, angle, and design) is problematic throughout most Australian jurisdictions [43], making camera detection of the signs difficult and, at times, impossible [44,45]. A recent review of speed management information in Australia identified that speed limit information is *widely utilised, diversely managed, dependent on legislative requirements and organisational structure, and spread throughout jurisdictions*, with limited guidance available at a national level for jurisdictions on speed management [46]. Regional and remote areas in Australia are particularly problematic, since the presence of speed signs is sporadic at best and the lack of digital or cellular connectivity in these areas means that even if vehicles are fitted with ISA, the technology will be of no use in assisting drivers to keep to speed limits. Ironically, regional and remote areas are also often responsible for a disproportionate number of deaths and serious injuries [47,48], and are thus likely to benefit most from having infrastructure that is supportive of driver assist technologies [49], including ISA. While digital HD speed data maps and GPS-linked speed limit databases are available, especially in urban city regions, limited cellular coverage across areas of Australia [40] means that camera detection of physical speed signs will currently be relied upon for ISA functioning in rural and remote areas. Speed signs are, therefore, a key input for sensing technology which allows ISA to function. In the absence of speed signs, on-board sensors, radars, and speed sign recognition cameras will not detect the data that are needed for ISA to be able to advise drivers of speeds or for ISA to regulate speed. Since speed sign availability is limited and, by law, the driver is often responsible for knowing the default speed in unmarked areas [50], the lifesaving potential of ISA in countries such as Australia may not always be realised and the benefits of ISA may potentially be overestimated.

### 1.2. Study Aims

Speed compliance encouraged through the use of ISA is expected to bring significant road safety gains in terms of speed-related crash mitigation. The overall objective of this study was to quantify the proportion of fatalities and serious injuries from ISA-sensitive crashes that will not be prevented due to the absence of speed signage. This required first quantifying the proportion of crashes that will likely be mitigated through speed compliance aided by ISA (independent of ISA-supportive infrastructure being present). Subsequently, the proportion of fatalities and serious injuries from speed-involved crashes that will not be prevented by ISA (i.e., the forgone savings) needed to be quantified, assuming that speed signage is the principal support for all vehicles fitted with ISA.

This objective was achieved by: (1) quantifying the proportions of crashes in Victoria, South Australia, and Queensland that were attributed to exceeding the speed limit (i.e., were ISA-sensitive) and the proportion of these that would be reduced by ISA based on technology effectiveness estimates published in the literature; (2) determining how many of these crashes occurred in areas that are not fitted with adequate speed signage; and (3) calculating the foregone saving of ISA in terms of crash-related injuries and fatalities and their correlated cost estimates. It is anticipated that the results of this study can inform appropriate investments into speed signage placement, or equivalent infrastructure, to ensure that ISA technologies perform as intended and provide life-saving benefits to road users.

## 2. Methods

Data on crashes reported by police from the Australian states of Victoria, South Australia, and Queensland were used in this study. Based on the assumption that crash trends and other parameters related to travel and driver behaviour remain similar over time and to ensure an adequate sample for analysis, data from 2013–2018 were used. The lost savings of ISA, assuming it were to be fitted to each vehicle, were estimated as follows:

1. Speed-related crashes (considered here to be *ISA-sensitive*) were identified from crash data (2013–2018 inclusive) using an established methodology [51] and mapped onto the road network with respect to remoteness level and road class.
2. ISA effectiveness estimates (published by Doecke, et al. [52]) were applied to ISA-sensitive crashes to quantify the number of fatalities and serious injuries likely prevented if ISA had been fitted to the crashed vehicles.
3. Using published literature that allowed speed sign availability to be approximated based on road class and remoteness level [53], ISA-sensitive crashes that would vs. would not be benefited by ISA were identified.
4. The lost benefits of ISA were quantified by estimating the number of fatalities and serious injuries that are unlikely to have been prevented due to the lack of speed signs at the crash locations.

**Figure 1:**
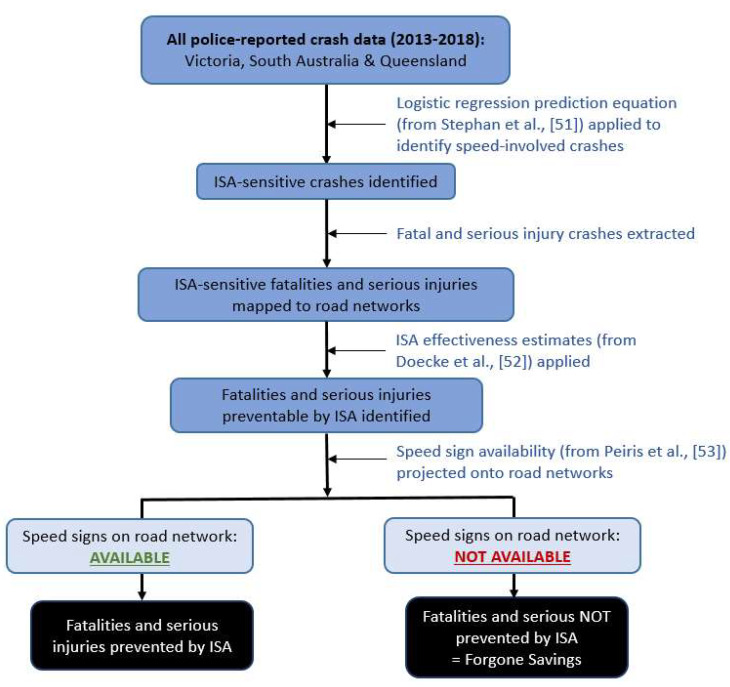
Schematic depicting the methodology which was undertaken to identify speed-related impacts that would be prevented by ISA and those which would not be prevented (forgone savings) due to the absence of speed signs.

A schematic summarising the overall methodology is presented in Figure 1, with each of the above steps outlined in greater detail below.

### 2.1. Identifying ISA-Sensitive Crashes from Crash Data

An analysis originally conducted in 2003 by Diamantopoulou, et al. [54] to determine the characteristics of speed-involved crashes, and updated in 2022 by Stephan, Budd, Newstead and Young [51], was used here to predict the ISA-sensitive crashes based on a number of available fields in the crash data. The re-analysis by Stephan and colleagues involved conducting a logistic regression using in-depth South Australian crash data [55,56,57] to identify factors associated with speed involvement in South Australian mass crash data (2008 to 2012). Stepwise logistic regression using likelihood ratio tests was used to identify main and interactive effects of model variables that contributed significantly to the model. The equation was generalised across locations, motor vehicle types, and speed zones to identify speed-involved crashes. The reader is referred to the study by Stephan, Budd, Newstead and Young [51] for an in-depth explanation of how the model was developed. For the current study, the prediction equation generated by Stephan, Budd, Newstead and Young [51] was applied to Victoria, South Australia, and Queensland mass crash data (2013–2018) to identify crashes likely to involve speeding.

There is some conjecture in the evidence regarding the contribution of speed in impacts [11,17,58,59,60,61]. The logistic regression prediction equation developed by Stephan, Budd, Newstead and Young [51] generates a probability of speed involvement for each crash. In doing so, every fatal and serious injury crash is ranked based on the model’s prediction of how likely each crash was to have been speed-related. In order to dichotomise the results and determine if a crash was speed-related or not, three specific thresholds were chosen: 10%, 15%, and 30%. While using a 10% threshold for speed involvement was likely to produce the best specificity for the speed prediction model, thresholds of 15% and 30% were also selected for greater sensitivity and to provide a range of positive predictive values. Due to the low specificity of the prediction equation (which generally produced very high probabilities of speed involvement across the crash population in each state), choosing three empirical cut-offs to dichotomise crashes as being speed-related or not provided a realistic range of outcome estimates. The most conservative estimate was used for the cost-benefit analysis.

In order to identify the speed-related crashes that were likely to be influenced by speed sign availability, the crashes in each state that were identified as being speed-related were plotted onto the road network using ArcGis Pro software. The crashes were then disaggregated by road class specific to each state and the remoteness levels across Australia, these being Major Cities, Inner Regional Australia, Outer Regional Australia, Remote Australia, and Very Remote Australia according to the Aria Index [62].

### 2.2. Applying ISA Effectiveness Values to the Crash Data

A literature review was conducted to identify the most suitable estimates of ISA crash reduction effectiveness. Since the benefits and limitations of ISA are likely to be country-specific and Australian crash data were used here to identify ISA-sensitive crashes, only ISA effectiveness values derived from Australian-run ISA trials using light vehicles were reviewed.

The effectiveness values for advisory ISA used in this study were derived from the 2009–2010 ISA trial funded by NSW RTA [52], in which 104 light vehicles were fitted with advisory ISA and speed data recorders. As part of the 3-month trial, vehicles communicated remotely with a centralised database so that up-to-date speed, location, and speed zone information could be constantly relayed. The road network within the trial area extended approximately 2500 km and incorporated more than 4000 speed signs across 932 speed zones, ensuring speed sign availability for ISA function. Baseline speed compliance of drivers prior to ISA installation was measured using a GPS-based speed and location data recorder fitted to all study vehicles at least one month prior to ISA device installation, logging the speed and location of each vehicle every ten seconds and producing a total of 7.5 million speed records (with the speed behaviour of drivers being measured before, during, and after the ISA device was operating in the vehicle). Modelling based on the results of this trial (using baseline speed compliance measurements) suggested that fatal and serious injuries in NSW would be reduced by 8.4% and 5.9%, respectively, if all vehicles in NSW were fitted with the technology. The reader is referred to Doecke, Kloeden and Woolley [52] for a detailed overview of the study, results, and analysis.

Since ISA changes the shape of the distribution of speed, using a speed-specific risk curve applied to the distribution of speeds in different speed zones allows the change in risk associated with speed distribution changes resulting from ISA fitment to be more accurately quantified. The reduction in crash risk using this approach was estimated by Doecke, Kloeden and Woolley [52] by applying crash risk curves for travel speeds originally generated by Kloeden, McLean, Moore and Ponte [56] in 1997, and developed further by the researchers in 2001 [57] and 2002 [63], to the speed distributions generated from the trial. Using the curves, the casualty crash risk reductions estimated for each speed zone were multiplied by the average number of fatal and serious injury crashes (2004–2008) within each speed zone across the Australian states [64] in order to determine annual crash reductions that could be achieved if all vehicles in Australia were fitted with advisory ISA.

The ISA effectiveness estimates generated by Doecke, Kloeden and Woolley [52] were applied in the current study to the fatalities and serious injuries resulting from the subset of crashes identified as being speed-involved (see Table 1 below).

### 2.3. Quantifying the Foregone Savings of ISA

As a premise for the calculations made in this study, it was assumed that, in Australia, the benefits of ISA will not be realised in regions where speed signs are unavailable. Current ISA technology fitted to new vehicles is highly reliant on speed sign recognition cameras communicating detected speed limits to drivers.

In order to approximate where ISA was most likely to function or fail based on speed sign availability, a methodology study was conducted in 2021 [65] to estimate speed sign availability in Victoria. In brief, this involved obtaining crash location data from respective state authorities to identify where (using GPS coordinates) each crash occurred. Fatal and serious injury (FSI) crashes were then mapped in Victoria (2013–2018) according to road class and remoteness level (as defined by the ABS 2016 [56]). Based on this distribution, locations were randomly selected to check for speed sign availability. Ten random locations (if <5% of FSI crashes occurred on the particular road class and remoteness level) were selected or twenty random locations (if >5% of FSI crashes occurred on the particular road class and remoteness level) were selected for the assessment per road class and remoteness level.

Speed sign data generated and made available by Data VIC [66] were overlaid on the Victorian road network map in ArcGis Pro software, and the distance from each random point to available speed signs was measured for both directions of travel in ArcGIS and verified using Google Maps. The distances were then averaged for each road type and remoteness level to identify the availability and frequency of speed sign locations based on road class and remoteness level. It was assumed that, in the absence of digital speed maps, drivers would have to travel these distances on average before vehicle-mounted speed sign detection cameras could identify a speed sign and inform drivers of the appropriate location-specific speed limit. The results of this analysis can be found in Peiris, Berecki-Gisolf, Newstead, Chen and Fildes [53].

Based on the findings from the methodology study [53], it was assumed that if 50% or more of the random locations identified did not have a speed sign available on the roads in that road class and remoteness level, speed sign availability for that road class and remoteness would be considered largely unavailable. Therefore, a vehicle travelling above the speed limit on that class of road would not benefit from ISA in terms of reduced crash risk or severity. Based on this assumption, six classes of road (disaggregated by remoteness level) were assumed to have no speed sign availability in Victoria. Since the road class hierarchy is similar in South Australia, the same assumption was made regarding speed sign availability across the road classes and remoteness levels (Table 2). In Queensland, however, since there are only five major road classes, a conservative approach was used to estimate speed sign availability. In Queensland, four classes of roads, that are each roughly equivalent to those in Victoria and South Australia, were assumed to not have speed sign availability (Table 3).

ISA-sensitive crashes that occurred on road classes where speed signs were not available, as identified in Table 2 and Table 3, were assumed to not benefit from ISA technology; this information was used to calculate the estimated potential forgone savings of ISA.

When estimating the economic benefits and forgone savings due to ISA, one of two approaches can be used to value the economic cost of road crashes (these being the willingness-to-pay and human capital approach). Here, a hybrid of the approaches (hybrid human capital), derived by BITRE [67], was used to estimate cost of death and injury from forgone ISA-sensitive crashes.

## 3. Results

The total number of fatalities (F) and serious injuries (SI) resulting from crashes reported to police over the six-year period analysed, and the subset of those where speed was identified as a likely causal factor using the logistic regression prediction equation developed by Stephan, Budd, Newstead and Young [51], is presented in Table 4.

Applying the estimated percentage (%) of casualty crash risk reductions per year produced by advisory ISA, as derived by Doecke, Kloeden and Woolley [52] and summarised by Wall, Creef, Boland, Vecovski, Prendergast, Stow, Fernandes, Beck, Doecke and Woolley [64], to the crashes identified as resulting from excessive speed, the safety benefits likely to be observed are summarised in Table 5. These estimates are based on the assumptions that all vehicles are fitted with advisory ISA, that the effectiveness rates specified are accurate, that ISA is functional in all instances, and that ISA is equally effective in reducing speeds amongst all driver demographics (i.e., comparable levels of speed compliance to those observed from the NSW trials), independent of crash location or circumstance.

Based on the generalised assumptions regarding speed sign availability across road classes and remoteness levels (Table 3 and Table 4), the lost benefits of ISA were estimated for each of the probability thresholds in each state (Table 6). That is, speed-related crashes that occurred on local roads in major cities, sub-arterial and local roads in regional areas, and sub-arterial, collector, and local roads in remote areas within Victoria and South Australia; and in Queensland, speed-related crashes that occurred on streets in major cities and regional areas, and streets and local connector roads in remote areas, were assumed not to be influenced by ISA due to the absence of speed signs.

Table 7 presents the estimated contribution of ISA to overall road safety in the three states in terms of the percentage of fatalities likely or unlikely to be prevented (due to absent speed signage) based on average annual road tolls (2013–2018). The 15% threshold estimates of ISA effectiveness were used to quantify the likely benefits of ISA on the overall road toll (measured in terms of fatalities). It is estimated that between 1.7 and 2.6% of the total road toll will continue to occur in each state because ISA will not be able to prevent these speed-related crashes due to absent speed signs.

### Estimating Economic Benefits and Forgone Savings Due to ISA

In the absence of a nationally agreed method for valuing road trauma, the Australian Government Office of Best Practice Regulation (OBRP) more recently published an updated means of estimating the base costs of road trauma. This method, *willingness-to-pay*, which is based on values of statistical life and statistical life year, was used by Economic Connections and published by the Australian Automobile Association (2017) to value the cost of a fatality at AUD 4.339 million and a serious injury at AUD 239,000, indexed to the year 2015 [68].

The monetary values of deaths and serious injuries estimated to be saved (due to functioning ISA) and foregone (due to ISA not being able to function in the absence of speed sign infrastructure) were expressed in monetary terms by multiplying the expected crash savings by the dollar values estimated for a fatality and serious injury by Economic Connections [68] (Table 8).

Estimates suggest that, in Victoria alone, deaths and serious injuries costing AUD 40 to 69 million annually could potentially be prevented by placement of speed sign infrastructure. These estimates are proportionately less in South Australia. In Queensland, lost lives and serious injuries resulting from high-speed crashes which could have been prevented by ISA would cost close to an estimated AUD 30 to 76 million.

## 4. Discussion

In the absence of cellular connectivity, speed sign sensing technology is solely reliant on speed signs being readily detectable. This study set out to estimate the lost benefits of ISA technology in three Australian states due to the absence of adequate speed sign infrastructure. By estimating the proportion of roads that are unlikely to support ISA (due to inadequate speed sign availability), and hence unlikely to prevent ISA-sensitive fatalities and serious injuries, the forgone benefits of ISA were estimated. It was assumed that roads that did not have speed signage or up-to-date digital speed maps that vehicles could access were correctly identified and would produce no road trauma savings for ISA-equipped vehicles. To do this, the presence and frequency of speed signs across road classes and remoteness levels in Victoria (based on a publicly available digital speed sign map) were quantified in a previously published desktop study [53], and these results were projected to South Australia and Queensland based on broad assumptions regarding speed sign availability across road class and remoteness levels. The number of fatalities and serious injuries that ISA may be able to mitigate was estimated by applying the most relevant advisory ISA effectiveness estimates available to speed-related (i.e., ISA-sensitive) fatal and serious injury crashes identified using historical crash data (disaggregated by road class and remoteness levels). Despite the fact that a large proportion of individuals suffer minor injuries in speed-involved crashes [69], and that ISA is likely to reduce these injuries, estimates of minor injuries prevented by ISA were not derived here due to minor injury data not being reliably collected or routinely validated within crash databases [11,58,60]. Therefore, the lost savings of ISA were calculated as lives lost and individuals seriously injured in speed-related crashes where ISA may have been of assistance in mitigating the crash but, ultimately, would not have been due to absent speed signage.

The estimates made in this paper regarding the benefits of ISA (and therefore the forgone savings) were based on a number of assumptions. These include that all light vehicles will be fitted with ISA in future and that crash trends remain similar over time across jurisdictions. Further, it was assumed that ISA is equally effective at reducing speeding amongst all driver demographics for the following reasons: (1) ISA fitment will induce comparable levels of driver compliance across the jurisdiction to those measured in the NSW ISA trial; (2) ISA functionality will be dependent on physical speed sign infrastructure; and (3) speed sign infrastructure remained unchanged over the study period. To our knowledge, no researchers to date have published a means of quantifying the ADAS technology benefits that are potentially foregone due to poor or absent road infrastructure. This work provides a novel contribution to efforts to better prepare roads for vehicles fitted with lifesaving ADAS technologies, such as ISA.

### 4.1. Effectiveness of ISA

Based on the stated assumptions, advisory ISA was estimated to potentially have the capacity to annually prevent 21–30 fatalities and 128–288 serious injuries in Victoria, 7–8 fatalities and 12–35 serious injuries in South Australia, and 18–27 fatalities and 121–320 serious injuries in Queensland. However, due to speed sign absence, approximately 28% of the fatal and serious injuries that ISA would be able to prevent in Victoria would not be prevented. Projecting the speed sign availability assumptions to South Australia and Queensland (based on road class and remoteness level), it was approximated that 29–34% of fatalities and serious injuries in South Australia and 27% of fatalities and serious injuries that could be prevented by ISA in Queensland would not be prevented. Annually, across the three states alone, it was estimated that between 11 and 15 lives would be lost and between 74 and 179 serious injuries would continue to occur in speed-related crashes that would otherwise be preventable with advisory ISA. These figures were not projected to a national level due to the inability to project assumptions regarding speed sign availability across road classes in other states for lack of comparative data. However, it is likely that the forgone benefits of ISA will be significantly higher in states that have more regional and remote areas of road travel exposure than Victoria does. The monetary values of the lost benefits of ISA, per annum, vary from AUD 30 to 69 million in Victoria and AUD 29 to 76 million in Queensland, though in South Australia they are proportionately less. Determining the percentage of total road trauma forgone nationally due to the absence of speed signage would be informative and help better direct infrastructure resources to regions where speed-related crashes are frequent but where ISA would be incapable of assisting drivers to be speed compliant.

While these figures should be interpreted with caution due to the number of assumptions made during the analysis, it is likely that the estimated foregone trauma savings due to absent speed signs are an underestimate of actual losses due to a number of factors including:

- The conservative estimates of crashes that were considered likely to involve speeding;
- The effectiveness rates of ISA employed here;
- Potential overestimates of speed sign availability in South Australia and Queensland since Victoria was used in the original research to generate assumptions about speed sign availability. Victoria may have more speed signs per road class than the other states.

### 4.2. Estimates of Speed Involvement in Crashes

The probability that a vehicle was exceeding the speed limit prior to impact was estimated using a logistic regression prediction equation [70]. While there are a number of limitations associated with the model (discussed below), the ranking and selection process is likely to have resulted in conservative estimates of speed involvement.

The logistic regression equation used in this study was based on in-depth South Australian crash data, which had been re-analysed and applied to Victorian mass crash data to identify factors associated with speed involvement. As such, there are a number of limitations associated with this model. The model was developed using SA in-depth crash data, which was restricted to casualty crashes that occurred in 60 km/h zones in urban areas involving passenger vehicles travelling at free speed for 1995 to 1997 [55,56]. The model developed based on this data was applied to all Victorian crashes to identify driver and vehicle characteristics associated with speed involvement (based on crash data from the years 2008 to 2012). Implicit is the assumption that factors that were associated with speed-involved passenger vehicle crashes in 60 km/h urban areas in South Australia were equally generalisable to Victorian crashes across all areas, speed zones, and vehicle types. As a consequence, it is likely that speed-related impacts, that are typically over represented in remote and regional areas of Australia [47], are under-represented in the number of fatal and serious injury crashes that were identified as being speed-involved in this analysis.

The application of this model to crash data from a different time period (2013–2018) and different state (such as Queensland) has not been validated. While results from the model should be interpreted with caution, three thresholds for identifying the probability of speed involvement were used. Based on the thresholds used, 19–40%, 15–42%, and 13–32% of fatal and serious injury crashes in Victoria, South Australia, and Queensland, respectively, were estimated to involve excessive speed as a crash-contributing factor. These figures are comparable to those published in the literature suggesting that, in New Zealand for example, close to 30% of fatalities and 20% of serious injuries are likely to involve excessive speeds (based on police reported crash data) [11]. Doecke and Woolley [71] identified that, in Victoria, over 30% of fatal crashes were likely to be speed-related (based on crash data), while in South Australia close to 40% of fatal crashes were speed-related. In Queensland, it was estimated that 22% of fatal crashes and close to 7% of serious injury crashes involved excessive speed, although the authors noted that limitations in data recording were likely to make these estimates conservative. When Event Data Recorder (EDR) data from South Australia were used to estimate the contribution of speed to fatal and serious injury crashes more recently, it was estimated that 18% of all fatal and serious injury crashes were a result of speeding [16]. Therefore, the estimates of speed-related fatal and serious injury crashes generated using the logistic regression equation here appear reasonable.

### 4.3. The Effectiveness Rates of ISA

**Figure 2:**
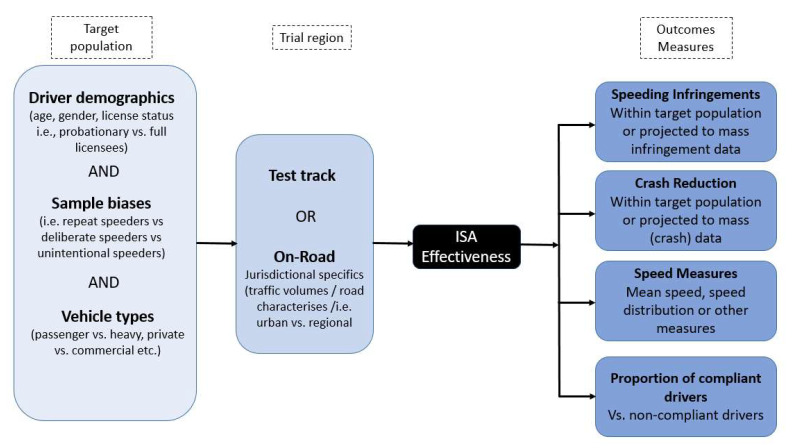
Schematic of how outcome measures of ISA effectiveness are influenced by the target population and trial region in ISA trials.

It is important to note that outcome measures or effectiveness estimates derived in ISA trials vary widely in terms of study settings, methodology, and results. For example, the speed reduction effects of ISA can be used to calculate the reduction in crash likelihood and severity. Using Nilsson’s [20] or Elvik’s power model [6], the changes in mean speed induced by ISA can be related to changes in crash frequency. Alternatively, Kloeden’s relative risk curves [55,56,57] can be used to predict the difference in casualty crashes for a change in the speed distribution (across speed zones) induced by the ISA technology. ISA effectiveness can also be reported in the form of other measures, such as time spent exceeding the speed limit, maximum or mean speeds pre-ISA vs. during-ISA, or numbers of speeding infringements issued pre-ISA compared to during or post-ISA. Ultimately, however, ISA effectiveness (independent outcome measures reported) is dependent on the pre-ISA travel speeds of the subjects taking part in the trial (which, in turn, depend on where the trial was conducted, i.e., on-road vs. test track, rural vs. urban, etc.), the demographics of the participants and selection biases, and the types of vehicles that the ISA devices were fitted to during the trial (i.e., private vs. commercial). ISA fitted to a less speed-compliant group of drivers who engage in inadvertent speeding, for example, is likely to have a more pronounced effect on mean travel and speed distributions and, therefore, provide a higher estimated benefit [72]. Factors that are likely to influence the estimated effectiveness of ISA are presented schematically in Figure 2.

Estimates of ISA effectiveness in terms of crash risk reduction generated from mean speed measures are readily available in the literature [73,74]. However, these estimates imply that ISA has a uniform effect on the reduction of speeds across all speed zones. The ISA effectiveness estimates used in this study were generated from the reduction in speed distribution attributed to ISA during the NSW ISA trial [64] (Table 1), similar to effectiveness estimates cited in other studies [75,76]. The magnitudes in crash risk reduction estimates generated by Wall, Creef, Boland, Vecovski, Prendergast, Stow, Fernandes, Beck, Doecke and Woolley [64], and used here, are comparable to crash reduction estimates cited in a number of other similar ISA trials run globally. For example, the long-term use of supportive ISA systems has been estimated to reduce injury crashes by up to 25% and fatal crashes by up to 32% [77], limiting ISA has been estimated to be up to 26.5% and 28.3% effective in reducing serious injury and fatal crash risk, and advisory ISA has been estimated to be up to 8.3% and 11% effective for reducing serious injury and fatal crash risk, respectively [76].

An independent investigation by TRL [12,78] suggested that, on average, 19% of fatalities would likely be prevented with the introduction of mandatory ISA (which could be over-ridden by applying the accelerator pedal). Carsten and Tate [32] estimated that fitting a *simple* mandatory ISA system to every vehicle would reduce injury crashes by 20% and fatal crashes by 37%, but a more advanced system capable of communicating dynamic speed changes via cellular connectivity would reduce injury crashes by 36% and fatal crashes 59%. Not surprisingly, the estimated crash reduction benefits of advisory ISA are typically less due to the less intrusive nature of the technology.

It is also noteworthy that the crash risk reduction estimates generated by Wall, Creef, Boland, Vecovski, Prendergast, Stow, Fernandes, Beck, Doecke and Woolley [64], and used here, were generated based on the change in the distribution of speeds (i.e., before vs. during ISA installation). Kloeden’s risk curves [56] relative to travel speed were used by the authors in the ISA study to estimate the reductions in crash risk. The equations for risk relative to travel speed (generated for 50 km/h and 60 km/h speeds) and mean speed (for 80 km/h or above) were applied to the speed distributions observed. While these risk curves are widely accepted, have been previously used for identifying the benefits of ISA [52,75], and are based on Australian conditions, there are limitations that may have affected the ISA effectiveness estimates derived from the NSW ISA trial. For example, Kloeden’s risk curves [56] may not apply to the full range of speed zones, environments, or driving/road conditions that subjects in the ISA trial were exposed to. Further, Kloeden’s et al.’s research used free travel speed to estimate crash risks, but this may not have been the case when applying the curves to the speed data generated from the ISA trial. More broadly, the observed speeding behaviour from the NSW ISA trial may not directly translate to Victoria, Queensland, or South Australia (to which the effectiveness values were applied), in which case ISA may not have an equivalent effect to that observed and quantified from the ISA trial. Further to this, the distribution of crashes per speed zone in Victoria, South Australia, and Queensland in the crash data which are used here may not be the same distribution of crashes per speed zone in the crash data that were used by the NSW ISA trial researchers to derive the effectiveness estimates.

Given that low-level speeding is socially acceptable in Australia and globally [1], it is difficult to accurately quantify the difference ISA will make to road safety. Driver acceptance (including usability) of ISA was not considered in this study. The actual effectiveness of ISA is likely to be highly dependent on how receptive or reactive drivers in future will be to warnings issued when ISA is functional. With advisory ISA, drivers are not obliged to reduce their speed when speed warnings are issued, and hence the level of speed compliance induced by advisory ISA is uncertain. When the beliefs underlying intensions to override ISA (to exceed the speed limit) were investigated using the Theory of Planned Behaviour, Rowe, et al. [79] found that driver attitudes strongly predicted these intentions. While the behavioural response of drivers to ISA is not within the scope of this study, it is important to state that since only advisory ISA was considered here, there is potential for drivers to ignore ISA-issued warnings and for the crash benefits (and therefore foregone benefits) of ISA to be severely overstated. Effectiveness estimates of limiting or mandatory ISA, therefore, are likely to be more reflective of actual ISA effectiveness for these ISA types, since this removes the behavioural or decision-making component associated with the technology. Data from international trials of supportive ISA systems suggest that speeding can be reduced by up to 53%, with potential reductions in serious injury crashes of up to 25% [80], or 50% if limiting ISA is considered [32]. However, given the European mandate for advisory ISA, only the forgone benefits of advisory ISA were considered here; therefore, effectiveness rates of advisory ISA were employed for this study. It is also noteworthy that, even if ISA is effective in encouraging speed compliance, limitations in driver reactions may, in some settings, only reduce the severity of a crash rather than prevent a crash. Further to this, ISA may be more effective in certain speed zones than others, as demonstrated across all of the Australian-run ISA trials [51,64,72,76]. Despite this, and the likely institutional and regulatory barriers associated with mandating the technology, ISA has the potential to encourage speed compliance and improve speed-related behaviour. The long-term benefits of deploying ISA, however, can only be realised if the infrastructure that supports its function is also made available.

### 4.4. Availability of Road Sign Infrastructure

In Australia, other than in the state of Victoria, road asset data pertaining to the availability of speed signs are not publicly available. It is therefore difficult to quantify the density of available speed signs across the nation. The availability of speed signs in Victoria was quantified in a previously published study based on a sampling methodology reflective of the distribution of FSI crashes [53]. Here, it was assumed that the same speed sign availability trends would be present across road classes and remoteness levels in South Australia and Queensland. This was considered to be a conservative means of gauging speed sign availability, since the position or visibility of the speed signs were not considered. That is, provided a speed sign was present, it was assumed that it would be detected by speed sign recognition cameras. While this method is a not a scientifically rigorous means of quantifying speed sign availability in the other states, in the absence of speed sign data, the projection allowed for speed sign availability to be estimated in order to quantify the readiness of roads for ISA.

To better understand the required investment in speed signage, in context of the potential cost saving from ISA, estimates are made of costs associated with installing speed signs across the road classes and remoteness levels deemed to have inadequate speed signs in this analysis (see Table 2 and Table 3). Assuming that these road classes have no speed signs at present, then the cost of installing new speed signs at 5 km intervals within major Cities, 15 km intervals in regional areas, and at 20 km intervals within remote areas would amount to approximately AUD 3.3 million in Victoria, AUD 4.0 million in South Australia, and AUD 3.7 million in Queensland. These costs are based on current estimates of installing a sign, pole, and foundation (including installation/labour) in Victoria. Assuming no maintenance or on-going costs are associated with the upkeep of static speed signs, this results in a one-off AUD 11 million investment across the three states to prevent annual losses (due to death and serious injury associated with road trauma) of approximately AUD 62 million. A 2011 analysis of costs associated with the upgrade and maintenance of the digital road network (again using Victoria’s cost estimates as the basis and projected nationally) suggested that it would cost approximately AUD 15.6 million to digitally map speeds in all Australian states and require a further AUD 2.4 million per year to update and maintain the maps (accounting for inflation, this is AUD 19.7 million and AUD 3 million, respectively, at current currency values). Given the somewhat restricted cellular connectivity throughout the nation, relying on digital speed maps alone for assisting drivers in being speed compliant is unlikely to be best practice, and investments into the strategic placement of static speed signs are recommended.

The assumption in this study that digital speed maps will have no effect on improving ISA function is unlikely to adversely impact the findings made here. While digital speed maps are available in most major city areas, and connectivity in these regions is high, changes in speed limit due to weather, road works, or traffic incidents are unlikely to be updated frequently enough for ISA systems to observe updated speed limits. Therefore, at present, speed signs will be heavily relied upon for adequate ISA function.

### 4.5. Future Research

This study was a macro-level analysis of the availability of physical speed signs (which constitute the required input data for onboard sensors and speed recognition cameras which, in turn, determines ISA function). The findings from this study suggest that the availability of speed signs in Victoria, and assuming similar speed sign availability in South Australia and Queensland, is inadequate to fully support advisory ISA in the absence of cellular connectively. Future research is recommended to identify speed sign availability in other states in order to determine if equal investments in infrastructure are necessary for the full potential of ISA to be realised across Australia. The effect that ISA is likely to have on mitigating minor injuries (not taken into account here) should also be considered so as to more accurately justify the investments that should be made across road networks to support ISA. Further, extending the scope of this work, the availability of cellular connectively can be taken into account so that the preparedness of the road network (for not only ISA but also for other autonomous vehicle technologies) can be evaluated, justifying more readily the investments in physical and digital infrastructure that should be made to gain maximum benefits from advanced driver assist technologies.

## 5. Conclusions

The annual forgone benefits of ISA, due to the absence of fixed speed signs estimated here, warrant investments into speed sign infrastructure, particularly since the annual economic costs of the lost benefits are likely to far outweigh that of one-off infrastructure investments in the installation of speed signs. In the interim, data collection and data sharing of current speed sign availability across Australian jurisdictions is recommended, as this will provide more realistic estimates of road-readiness for technologies such as ISA and allow for more targeted and strategic investments. Currently, the true extent of infrastructure deficiencies remains unknown. The estimates of the forgone benefits of ISA generated here use the best available data, a sound methodology, and a transparent approach. In doing so, the need for investments into speed sign infrastructure likely to bring significant additional road safely gains in terms of lives lost and injuries prevented is identified.

## Figures and Tables

**Table 1:** Estimated annual percentage (%) of reductions in excessive-speed crashes produced by advisory ISA based on crash data (2013–2018) using effectiveness estimates derived by Doecke, Kloeden and Woolley [52].

Crash Type	Victoria	South Australia	Queensland
Fatal	18.7	19.5	18.8
Serious injury	19.1	19.3	19.6

**Table 2:** Assumptions made regarding speed sign availability across remoteness levels (Major City, Regional, and Remote) and road classes in Victoria and South Australia.

Road Class	Major City	Regional	Remote
Freeway	✓	✓	✓
Highway	✓	✓	✓
Arterial	✓	✓	✓
Sub-Arterial	✓	🗶	🗶
Collector	✓	✓	🗶
Local	🗶	🗶	🗶

(✓) speed signs were considered to be available; (🗶) speed signs were considered to be unavailable.

**Table 3:** Assumptions made regarding speed sign availability across remoteness levels (Major City, Regional, and Remote) and road classes in Queensland.

Road Class	Major City	Regional	Remote
Freeway	✓	✓	✓
Highway	✓	✓	✓
Secondary	✓	✓	✓
Local Connector	✓	✓	🗶
Streets	🗶	🗶	🗶

(✓) speed signs were considered to be available; (🗶) speed signs were considered to be unavailable.

**Table 4:** Number of fatalities (F) and serious injuries (SI) from crashes identified as being speed-related. Results are shown using various thresholds (top 10%, 15%, and 30% of crashes) and ranked according to estimated likelihood of speed involvement.

		Total		F and SI in Speeding-Related Crashes at Various Thresholds					
		F	SI	F (10%)	SI (10%)	F (15%)	SI (15%)	F (30%)	SI (30%)
Victoria		1505	23,301	696	4024	757	5289	958	9048
South Australia		454	2701	135	364	152	565	246	1094
Queensland		1480	31,249	582	3726	678	5251	846	9784

**Table 5:** Estimated annual reductions in fatalities and serious injuries from excessive-speed crashes, as identified from crash data in each state, based on crash reduction estimates specified by Wall, Creef, Boland, Vecovski, Prendergast, Stow, Fernandes, Beck, Doecke and Woolley [64].

State	Threshold *	Total F and SI Prevented	Fatalities Prevented	Serious Injuries Prevented
Victoria	10	149.6	21.7	127.9
	15	191.7	23.6	168.1
	30	317.5	29.9	287.7
	10	16.1	4.4	11.7
South Australia	15	23.1	4.9	18.2
	30	43.2	8	35.2
	10	140.0	18.2	121.7
Queensland	15	192.8	21.2	171.5
	30	346.1	26.5	319.6

* Thresholds refer to the top 10%, 15%, and 30% of crashes most likely to have involved speeding based on the calculated probabilities.

**Table 6:** Annual fatalities (F) and serious injuries (SI) likely to be prevented and unlikely to be prevented in speed-related crashes (due to absent speed signs).

State and Threshold *		Total F and SI	F		SI		Total	
			Prevented	Not Prevented	Prevented	Not Prevented	Prevented (%)	Not Prevented (%)
Victoria	10	149.6	15.7	6.0	92.3	35.7	108.0 (72.2)	41.6 (27.8)
	15	191.7	17.0	6.5	121.0	47.0	138.1 (72.0)	53.6 (28.0)
	30	317.5	21.9	7.9	208.7	78.9	230.6 (72.6)	86.9 (27.4)
South Australia	10	16.1	3.2	1.2	7.4	4.3	10.6 (65.8)	5.5 (34.3)
	15	23.1	3.5	1.5	12.2	6.0	15.6 (67.7)	7.5 (32.4)
	30	43.2	5.9	2.1	24.9	10.3	30.8 (71.4)	12.3 (28.6)
Queensland	10	140.0	14.4	3.9	88.2	33.5	102.6 (73.3)	37.4 (26.7)
	15	192.8	17.0	4.2	123.5	48.1	140.5 (72.9)	52.3 (27.1)
	30	346.1	21.2	5.3	229.9	89.7	251.2 (72.6)	95.0 (27.4)

* Thresholds refer to the top 10%, 15%, and 30% most likely to involve speeding based on the calculated probabilities.

**Table 7:** Percentage of ISA-sensitive fatalities prevented and not prevented, with respect to the annual average road toll, due to the presence/absence of speed signs.

State	Road Toll (Fatalities)	Fatalities in ISA-Sensitive Crashes		% Road Toll	
		Prevented	Not Prevented	Prevented	Not Prevented
Victoria	251	17	6.5	6.8	2.6
South Australia	76	3.5	1.5	4.6	2.0
Queensland	247	17	4.2	6.9	1.7

**Table 8:** The estimated costs and foregone savings * (AUD million) due to fatalities and serious injuries resulting from ISA-sensitive crashes where ISA could have intervened (based on speed sign availability).

State and Threshold		Prevented		Not Prevented	
		F	SI	F	SI
Victoria	10	55.25	21.11	28.96	11.20
	15	59.82	22.87	37.97	14.75
	30	77.07	27.80	65.49	24.76
South Australia	10	11.26	4.22	2.32	1.35
	15	12.32	5.28	3.83	1.88
	30	20.76	7.39	7.81	3.23
Queensland	10	50.67	13.72	27.68	10.51
	15	59.82	14.78	38.75	15.09
	30	74.60	18.65	72.14	28.15

* All costs associated with death and serious injuries calculated here are expressed in 2022 AUD.

## Data Availability

Not applicable.
